# Supplementary material for: Multi-COBRA hemagglutinin formulated with cGAMP microparticles elicits protective immune responses against influenza viruses
Source: mSphere. 2024 Jun 26;9(7):e00160-24. doi: 10.1128/msphere.00160-24 (PMC11288037; doi:10.1128/msphere.00160-24)
Supplement: Fig S1 — Scanning electron micrographs of blank and cGAMP loaded Ace-DEX MPs. [file msphere.00160-24-s0001.pdf]

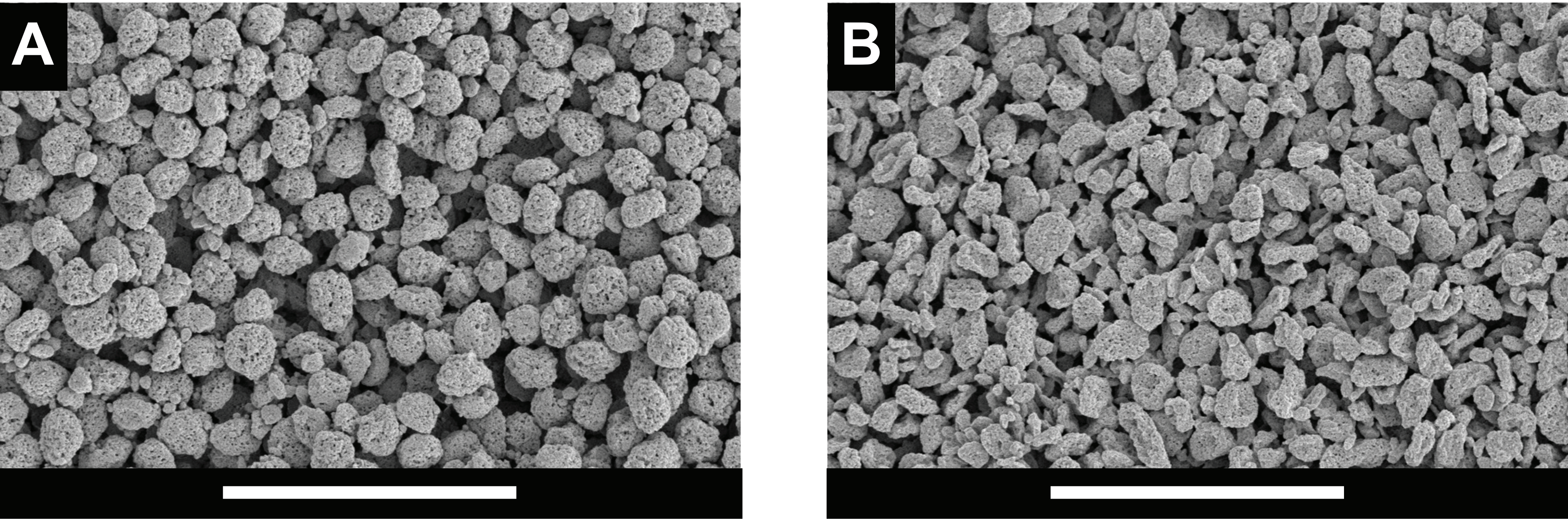

Supplementary Figure 1. Scanning electron micrographs of (A) blank Ace-DEX MPs and (B) cGAMP loaded Ace-DEX MPs prepared via the electrospray method. Scale bars represent 5 μm.
