# Supplementary material for: Multi-COBRA hemagglutinin formulated with cGAMP microparticles elicits protective immune responses against influenza viruses
Source: mSphere. 2024 Jun 26;9(7):e00160-24. doi: 10.1128/msphere.00160-24 (PMC11288037; doi:10.1128/msphere.00160-24)
Supplement: Fig S2 — Hemagglutinin inhibition assay H1 virus panel. [file msphere.00160-24-s0002.pdf]

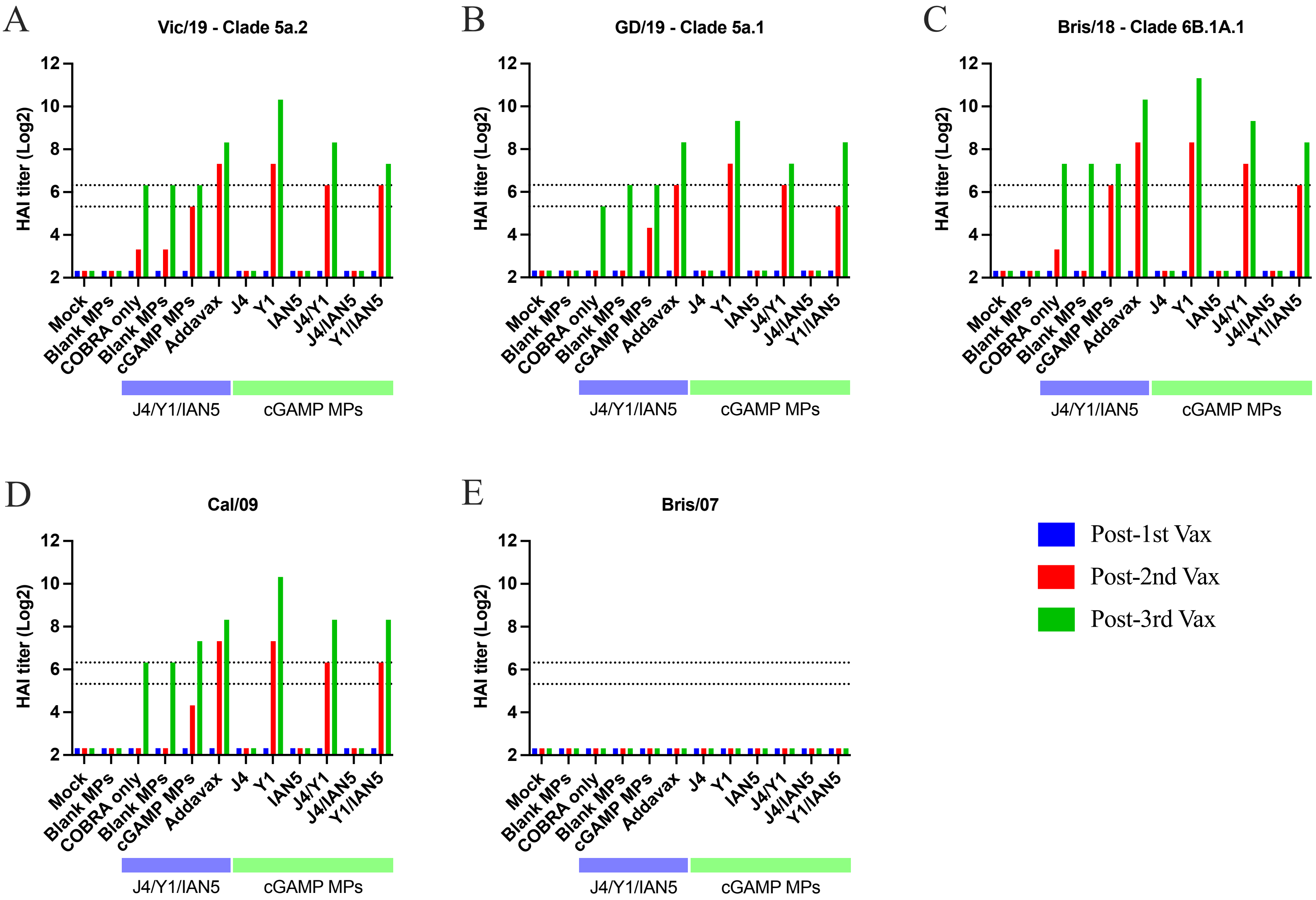

Supplementary Figure 2. Hemagglutinin inhibition assays H1 virus panel. Pooled mice serum collected after each vaccination were used in HAI assay against a panel of historical H1N1 influenza viruses. The title of each figure indicates the virus name. The x-axis indicates the experimental group. The y-axis indicates HAI titer in Log2. The lower dashed line indicates 1:40 and the higher dashed line indicates 1:80.
