# Supplementary material for: Multi-COBRA hemagglutinin formulated with cGAMP microparticles elicits protective immune responses against influenza viruses
Source: mSphere. 2024 Jun 26;9(7):e00160-24. doi: 10.1128/msphere.00160-24 (PMC11288037; doi:10.1128/msphere.00160-24)
Supplement: Fig S5 — Hemagglutinin inhibition assays. [file msphere.00160-24-s0005.pdf]

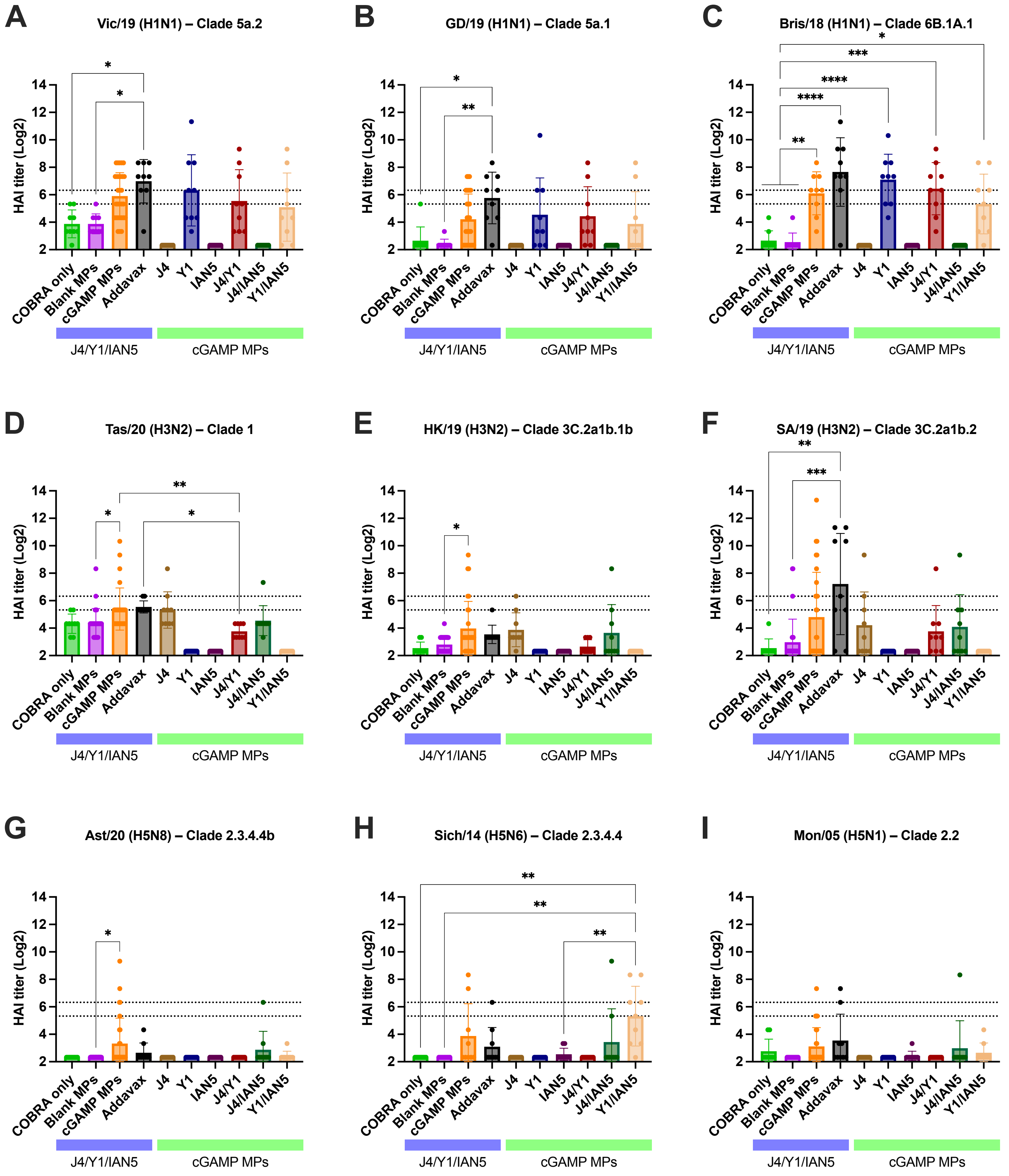

Supplementary Figure 5. Hemagglutinin inhibition assays. Individual mice serum collected after boost were used in HAI assay against a panel of historical H1N1, H3N2, and H5Nx influenza viruses. The title of each figure indicates the virus name. The x-axis indicates the experimental group. The y-axis indicates HAI titer in Log2. The lower dashed line indicates 1:40 and the higher dashed line indicates 1:80. HAI titers were statistically analyzed using nonparametric one-way analysis of variance (ANOVA). A P value of less than 0.05 was defined as statistically significant (*, $P < 0.05$; **, $P < 0.01$; ***, $P < 0.001$; ****, $P < 0.0001$). Data is presented as average ± standard deviation.
