# Supplementary material for: Multi-COBRA hemagglutinin formulated with cGAMP microparticles elicits protective immune responses against influenza viruses
Source: mSphere. 2024 Jun 26;9(7):e00160-24. doi: 10.1128/msphere.00160-24 (PMC11288037; doi:10.1128/msphere.00160-24)
Supplement: Fig S6 — Mice challenged with Bris/18 H1N1 influenza virus. [file msphere.00160-24-s0006.pdf]

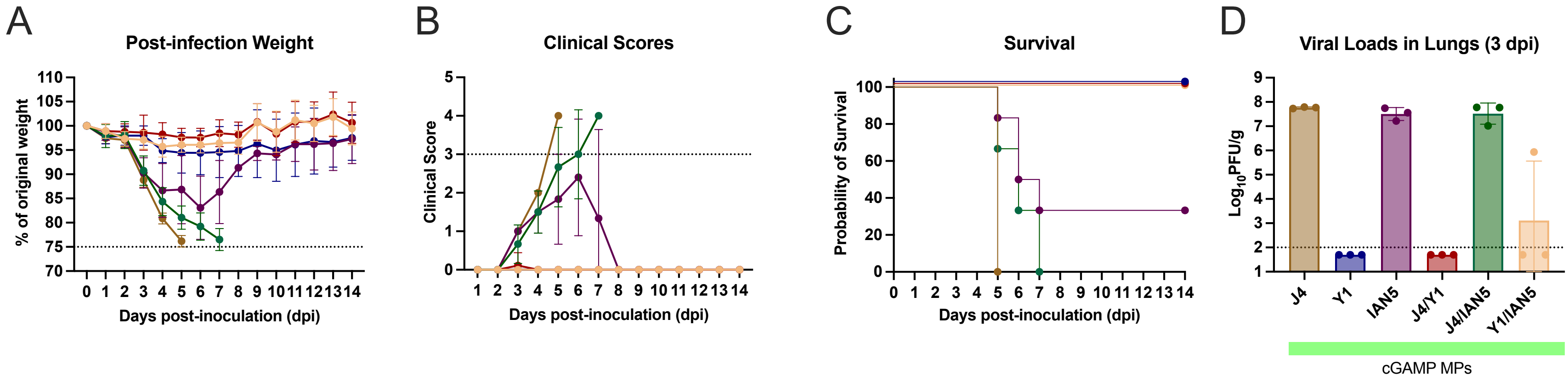

Supplementary Figure 6. Mice challenged with Bris/18 H1N1 influenza virus. (A) The weight loss curves, (B) survival, (C) clinical scores, and (D) pulmonary viral loads on day 3 post-infection. Colors indicate experimental groups given in D. Data is given as average ± standard deviation. Statistical analysis was conducted using nonparametric one-way analysis of variance (ANOVA). A P value of less than 0.05 was defined as statistically significant (*, P < 0.05; **, P < 0.01; ***, P < 0.001; ****, P < 0.0001).
